# Research on the emotional experience of online tourists in Zhangjiajie based on text mining and LDA-FDEMATEL

**DOI:** 10.1371/journal.pone.0355471

**Published:** 2026-10-01

**Authors:** Cheng-Ru Wu, Ming-Han Chiang, Bao-Hui Huang, Hung-Lung Lin, Yanran Huang

**Affiliations:** 1 College of Tourism Management, Wuyi University, Wuyishan, Fujian, China; 2 National Tainan Junior College of Nursing, Tainan City, Taiwan; 3 School of Economics and Management, Sanming University, Sanming, Fujian, China; Guilin University of Technology, CHINA

## Abstract

Online travel platforms generate extensive user-generated content (UGC), yet the causal structure of tourists’ emotional experiences remains insufficiently understood. This study examines Zhangjiajie Scenic Area by integrating Latent Dirichlet Allocation (LDA) with fuzzy DEMATEL and DANP to extract emotional themes and identify their hierarchical relationships within a destination system. Results reveal a three-layer structure of emotional experience formation: an input layer (management systems, service processes, and vegetation landscapes), a contextual conditioning layer (water, mountain, architectural landscapes, and temporal regulation), and an outcome layer (visitor activities, perceptions, evaluations, and impressions). These layers interact through a transformation process that shapes how environmental stimuli are converted into emotional responses. The findings demonstrate that emotional experience is systemically constructed through hierarchical causal interactions rather than isolated attributes. The study provides a structured framework for tourism emotion analysis and offers evidence-based implications for emotion-oriented destination management in nature-based tourism contexts.

## 1. Introduction

### 1.1. Research background

In the digital era, tourists increasingly share text, photos, and short videos in real time on social media and travel platforms to document their travel experiences and emotions [1]. This user-generated content (UGC) provides rich information on how tourists perceive natural and cultural landscapes [2,3] and express their emotional reactions [4,5]. As a result, UGC has become an important data source for understanding destination image formation and tourist behavior [6,7].

Zhangjiajie, a UNESCO World Geopark and one of China’s most internationally recognizable mountain destinations, is renowned for its dramatic sandstone-peak forest landscape. The site attracts large numbers of both domestic and international tourists and is widely known for its natural scenery. Its visually striking environment elicits strong embodied perceptions and emotional responses, making it a suitable context for studying mechanisms of tourism emotion. Insights derived from Zhangjiajie, therefore, offer implications for both Chinese scenic areas and global nature-based destinations. In the context of tourism innovation, converting UGC into actionable knowledge for experience management has become an important issue for both researchers and practitioners [8,9].

### 1.2. Research gaps

Although topic modeling techniques such as Latent Dirichlet Allocation (LDA) have been increasingly applied to extract tourists’ perceptions and emotions from UGC [10–14], several critical research gaps remain.

First, existing UGC-based studies primarily identify emotional themes but rarely examine structural or causal relationships among emotional factors. For example, Guo et al. (2017), Chen et al. (2021), and Huo et al. (2024) extracted emotional topics but did not analyze interdependencies or causal linkages [10,12,15]. This limits understanding of how emotional components interact within destination systems.

Second, prior studies often focus on isolated emotional constructs—such as immersive experience [16], cultural identity [17], or emotional framing [18]—without integrating them into a unified emotional mechanism, resulting in fragmented explanations of how tourist experience forms.

Third, most tourism emotion studies rely on correlation or regression-based methods [19,20], which cannot capture causal interdependence. Although fuzzy DEMATEL has been applied in engineering and sustainability research [21–23], its application in tourism emotion research remains limited, primarily confined to service quality or ecotourism assessments [24,25]. Consequently, the causal hierarchy of emotional experience in tourism contexts remains underexplored, mainly representing a substantial theoretical and practical gap.

### 1.3. Research objectives and questions

To address the research gaps identified above, this study develops a hybrid analytical framework that integrates text mining, LDA topic modeling, and fuzzy multi-criteria decision analysis. Fig 1 provides an overview of the research process, summarizing the sequential procedures from UGC collection and text preprocessing to topic extraction, expert interpretation, and the fuzzy DEMATEL–DANP analysis.

**Fig 1:**
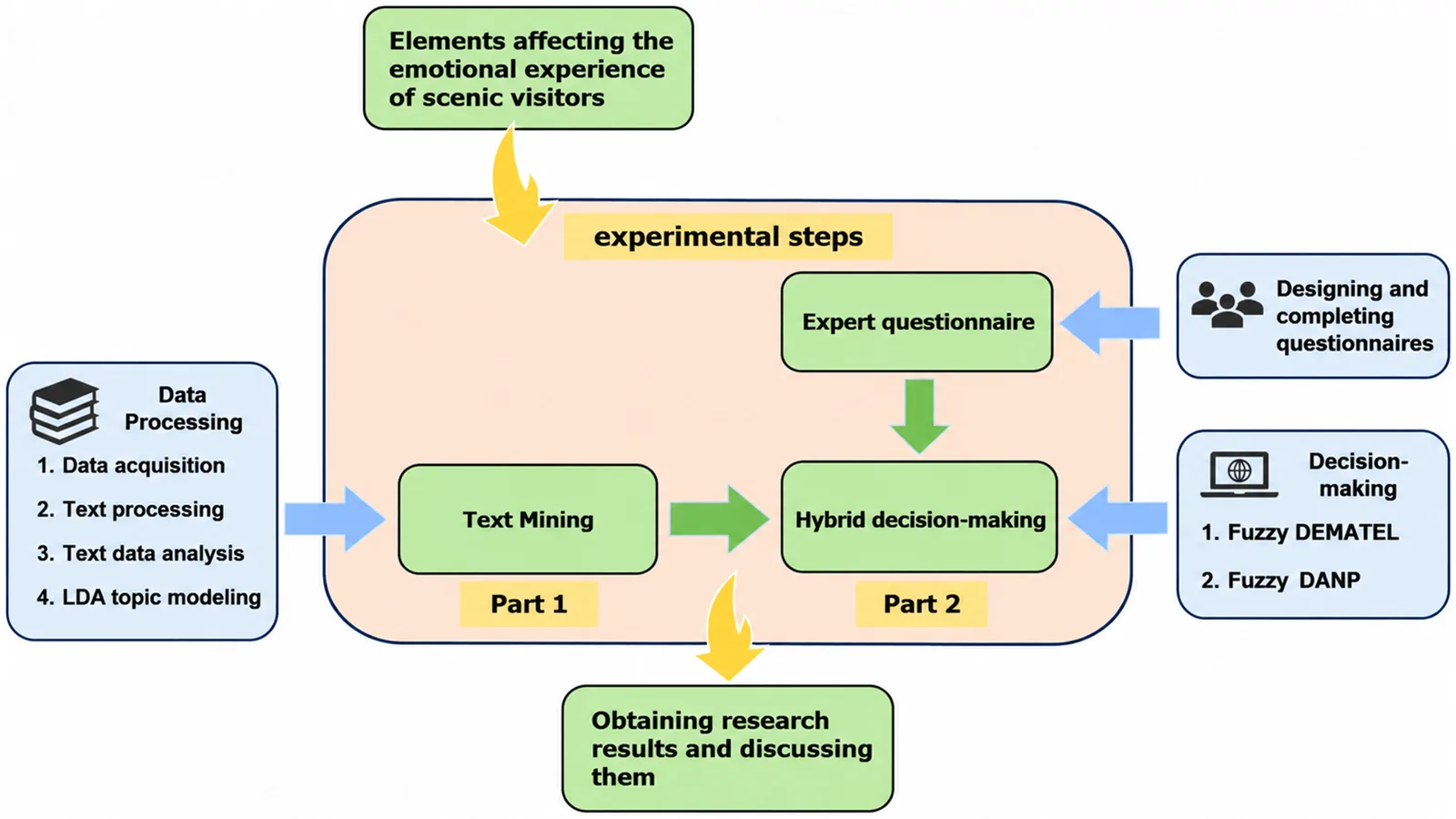
Flowchart illustrating the overall research process. The figure summarizes sequential steps, including data collection, preprocessing, LDA topic modeling, expert interpretation, fuzzy DEMATEL analysis, fuzzy DANP weighting, and result integration.

First, UGC related to the Zhangjiajie Scenic Area was collected from major travel platforms and then cleaned and preprocessed to extract tourists’ emotional expressions [7,26,27]. Second, the LDA model was employed to identify emotional themes and semantic patterns [12,14,28]. Based on topic keywords, an expert panel interpreted and defined emotional experience factors [7,29]. Third, fuzzy DEMATEL was used to identify causal relationships and the strength of influence among these emotional factors [22,23]. Fourth, fuzzy DANP was applied to calculate dependency-based weights and integrate the causal structure into a hierarchical evaluation framework [30,31]. Finally, the results were synthesized to identify key emotional drivers and derive management implications for enhancing visitor experience in Zhangjiajie [30,32].

Accordingly, this study addresses the following questions:

(1) What themes reflect tourists’ emotional experiences in UGC about Zhangjiajie?
(2) How are different emotional factors causally related, and what is the strength of their influence?
(3) Which emotional components play the most influential roles in shaping overall visitor experience?

### 1.4. research innovation and contributions

This study contributes to tourism research in three ways.

(1) Methodological contribution.

This study integrates LDA with fuzzy DEMATEL–DANP, extending UGC-based tourism research from descriptive topic identification to causal-structural analysis of emotional systems.

(2) Theoretical advancement.

This study refines the emotion–experience–behavior framework [33,34] and extends research on embodied emotional experiences in nature-based tourism contexts [35] by introducing a causal system perspective.

(3) Practical implications.

The findings provide actionable insights for destination management by identifying key emotional drivers that can support resource allocation, service coordination, and experience design in Zhangjiajie and similar destinations [34,35].

The rest of this paper is organized as follows. Section 2 reviews the literature; Section 3 presents the methodology; Section 4 reports results; Section 5 discusses findings; Section 6 concludes the study.

## 2. Research review

### 2.1. Emotional tourist experience: Theories, drivers, and research gaps

Tourists’ emotional experiences represent a core mechanism linking environmental stimuli to cognitive appraisal and behavioral responses in tourism settings [36], where emotions are not direct reactions but outcomes of evaluative psychological processes [7,37]. From the perspective of cognitive appraisal theory, emotional responses arise from individuals’ subjective evaluation of external stimuli, which are first perceived and cognitively interpreted before being translated into affective and behavioral responses [33,37]. Recent tourism psychology research further emphasizes that emotional experience is inherently multi-source and context-dependent, involving the interaction of environmental stimuli, cognitive interpretation, and behavioral intention formation processes [7,38].

While emotional experiences originate from cognitive appraisal processes, the stimuli shaping such evaluations are embedded in broader destination environments that integrate both natural and built elements, including landscapes, facilities, services, and management systems [39,40]. From a Resource-Based View (RBV) perspective, these natural and cultural resources constitute the structural foundation of destination competitiveness and the creation of emotional value [41–43]. However, RBV primarily explains structural endowments rather than experiential realization. Service-Dominant Logic (SDL) addresses this limitation by emphasizing that emotional value emerges through continuous interactions between tourists and destination systems, including service encounters and management processes [44,45]. Complementing this interactional perspective, Experience Economy theory further explains how emotional value is realized through immersive, aesthetic, and affective experiential dimensions that ultimately shape satisfaction and behavioral intention [34,46].

Despite these theoretical advances, existing empirical studies remain largely descriptive and component-based, focusing on isolated constructs such as experience quality, destination image, or emotional response without capturing their interdependence or systemic structure [13,47]. This limitation has been increasingly highlighted in recent tourism emotion research, which argues that emotional responses should be understood as structured outcomes that emerge from multi-layered cognitive–affective interactions rather than as independent variables [12,48]. Accordingly, the emotional experience of tourism can be conceptualized within a stimulus–organism–response (S–O–R) framework, in which environmental stimuli are first perceived, cognitively appraised, and subsequently transformed into emotional states and behavioral intentions [33,48]. However, despite this advancement, prior studies still fail to distinguish between causal drivers and outcome-level emotional components or to capture their hierarchical interdependencies within a unified system. Therefore, a system-level analytical framework is required to integrate semantic extraction with causal structure modeling. To address this gap, this study integrates LDA-based topic modeling with fuzzy DEMATEL–DANP analysis to systematically uncover the latent structure, causal hierarchy, and relative importance of emotional experience factors in tourism contexts.

### 2.2. UGC and LDA in tourism research

The rapid expansion of online platforms has positioned user-generated content (UGC) as a critical data source for understanding tourists’ perceptions, emotions, and behavioral patterns, and it has been widely used in tourism research to capture large-scale experiential narratives that reflect destination image formation and visitor experience heterogeneity [6,10,12,19,48]. To analyze such unstructured textual data, topic modeling techniques—particularly Latent Dirichlet Allocation (LDA)—have been widely adopted as an effective approach for identifying latent semantic structures within large-scale corpora [12,13]. In tourism studies, LDA enables the transformation of fragmented textual expressions into structured thematic representations by detecting probabilistic patterns of word co-occurrence, thereby facilitating the extraction of dominant experiential dimensions embedded in visitor-generated narratives [12,49,50]. Its robustness for large-scale semantic discovery has also been widely validated in social media and discourse mining research [14]. Beyond tourism, LDA has also demonstrated strong applicability in social media analytics and large-scale discourse mining, confirming its robustness for unsupervised semantic discovery across domains [51–54].

Despite its effectiveness in uncovering latent topics, LDA remains essentially a descriptive technique that focuses on semantic clustering rather than structural explanation. In tourism contexts, where experiential dimensions are inherently interconnected, the assumption of topic independence limits its ability to capture interaction effects, causal dependencies, and hierarchical organization among experiential components. As a result, LDA-derived outputs often reflect parallel thematic structures rather than an integrated system for the formation of tourist experience [12,49].

To address this limitation, this study integrates LDA with a fuzzy DEMATEL–DANP framework. LDA is first used to identify latent experiential themes in UGC, while fuzzy DEMATEL is employed as the core technique to uncover causal relationships and directional influences among these themes. Subsequently, DANP is applied to derive dependency-based weights, thereby quantifying both structural importance and hierarchical influence. This integrated framework enables a shift from purely semantic topic extraction to system-level causal structure modeling, offering a more comprehensive explanation of how tourists’ emotional experiences are formed in natural scenic destinations.

### 2.3. application of fuzzy DEMATEL and DANP in tourism research

Fuzzy DEMATEL is a structural analysis method designed to examine complex systems characterized by interdependencies, uncertainty, and feedback effects [23,24,26]. Incorporating fuzzy logic into the traditional DEMATEL framework enables the expression of expert judgments under uncertainty while capturing both direct and indirect influence relationships among variables [17,23,26,55]. These properties make it particularly suitable for tourism contexts, where emotional perceptions and experiential evaluations are inherently interdependent and dynamically formed. In tourism and hospitality research, fuzzy DEMATEL and its extended form (DANP) have been widely applied to analyze service quality systems, destination management structures, and operational interdependencies in complex tourism environments [3,24,31,56].

Recent methodological advances have further highlighted the value of integrating text-mining techniques with fuzzy multi-criteria decision-making approaches, particularly through combining LDA-derived semantic structures with causal modeling frameworks to enhance the explanatory power of large-scale textual data [30,57,58]. However, existing applications remain largely concentrated on service systems and operational efficiency, with limited attention to the structural organization of tourists’ emotional experience as an interconnected system [24,31,56]. In addition, most prior studies focus on isolated factors, failing to capture their hierarchical and causal dependencies within experiential narratives [6,12,13,49]. To address these limitations, fuzzy DEMATEL is employed to identify directional influence relationships among emotional dimensions, while DANP is used to derive dependency-based importance weights, enabling simultaneous assessment of causal strength and hierarchical importance within the emotional experience system.

## 3. Materials and methods

From the perspective of tourism emotional experience, this study applies text mining and the Latent Dirichlet Allocation (LDA) model to extract semantic themes from user-generated content (UGC) collected from Chinese online travel agency (OTA) platforms. LDA is employed to identify latent emotional and cognitive patterns embedded in unstructured textual data, thereby constructing an initial indicator system of tourism emotional experience factors for subsequent quantitative analysis.

### 3.1. Case study: Zhangjiajie tourist attractions

Zhangjiajie, located in Hunan Province, China, is characterized by quartz–sandstone peak forest geomorphology and complex karst landscapes. It comprises multiple core scenic areas, including Zhangjiajie National Forest Park, Tianzi Mountain, Yangjiajie, Suoxiyu, and Tianmen Mountain, as well as representative infrastructures such as the Bailong Elevator, Glass Skywalk, and Huanglong Cave. These natural and built environments jointly constitute a mixed landscape system that generates diverse tourist perceptions and experiential responses.

In recent years, Zhangjiajie has experienced sustained growth in tourism flows, supported by transportation improvements, infrastructure development, product diversification, and smart tourism applications. Correspondingly, large volumes of user-generated content (UGC) have emerged across OTA platforms, documenting visitors’ perceptions of landscape features, service encounters, and environmental conditions. This continuously generated textual corpus provides a suitable empirical basis for analyzing the structure of emotional experiences in tourism.

This study uses Zhangjiajie as a case context to construct a data-driven indicator system of emotional experience factors derived from large-scale UGC, providing a structured empirical basis for subsequent causal analysis of experiential dimensions.

### 3.2. Data collection and pre-processing

This study collected user-generated content (UGC) related to “Zhangjiajie” from three major Chinese online travel platforms: Ctrip, Tongcheng, and Dianping. Reviews were retrieved using the Octoparse crawler, covering the period from January 2019 to October 2024, spanning pre-pandemic, pandemic, and post-pandemic phases. To reduce temporal imbalance, a time-stratified sampling strategy was used to ensure proportional representation of reviews across years.

All data were collected from publicly available sources in compliance with the platform’s terms of service and used exclusively for academic research. Only publicly visible textual content was extracted. During preprocessing, all potential personal identifiers, including usernames, user IDs, profile information, hyperlinks, and other metadata, were removed to ensure full anonymization. No personally identifiable information was retained in the final dataset.

To ensure temporal robustness, a semantic stability test was conducted by dividing the corpus into three periods (2019–2020, 2021–2022, and 2023–2024) and examining the consistency of high-frequency terms across segments. Core experiential terms such as “scenery,” “cable car,” “queue,” and “service” remained stable across periods, indicating structural consistency in the expression of tourist experience over time. Texts unrelated to on-site experiences (e.g., ticket refunds, lockdown announcements, and flight disruptions) were excluded to reduce contextual noise.

The final dataset comprised 8,592 valid reviews, including 4,604 from Ctrip, 2,271 from Tongcheng, and 1,717 from Dianping. Text segmentation was performed using the Jieba library in Python, and stop words were removed using dictionaries from the Harbin Institute of Technology and Baidu. Word frequency statistics were computed using the *collections.Counter* module to support subsequent topic modeling analysis.

### 3.3. descriptive text analysis

Before topic modeling, a descriptive statistical analysis was conducted to examine the lexical characteristics of the processed corpus. Word frequencies were calculated using Python’s *collections.Counter*. Table 1 presents the top 27 high-frequency terms extracted from the dataset, which primarily include place names (e.g., Zhangjiajie, Tianmen Mountain), attraction-related terms (e.g., Scenic Area, Landscape), infrastructure-related terms (e.g., Cableway, Elevator, Glass), and operational experience terms (e.g., Queue, Ticket, Sightseeing).

**Table 1:** Top 48 high-frequency terms in Zhangjiajie reviews.

Keyword	Frequency	Keyword	Frequency	Keyword	Frequency
Zhangjiajie	8266	No	2640	Feeling	2003
Scenic Area	5338	Glass	2546	Elevator	1977
Good	3874	Convenient	2466	Hour	1941
Cableway	3748	Queue	2392	Place	1850
Very	3551	Time	2219	Experience	1707
Scenery	3309	National Forest Park	2113	Inside	1630
Attraction	3147	Really	2101	Many	1613
Landscape	2825	Ticket	2097	Downhill	1533
Tianmen Mountain	2757	Sky Ladder	2058	Sightseeing	1520

For reporting consistency, Chinese terms were translated into English, while all subsequent topic modeling analyses were conducted on the original Chinese corpus. A word cloud was generated using the *WordCloud* and *matplotlib libraries* to provide a visual representation of term distribution (Fig 2). The visualization reflects the frequency-based prominence of key lexical items within the corpus. Both the frequency table and the word cloud provide an overview of the lexical distribution prior to LDA modeling.

**Fig 2:**
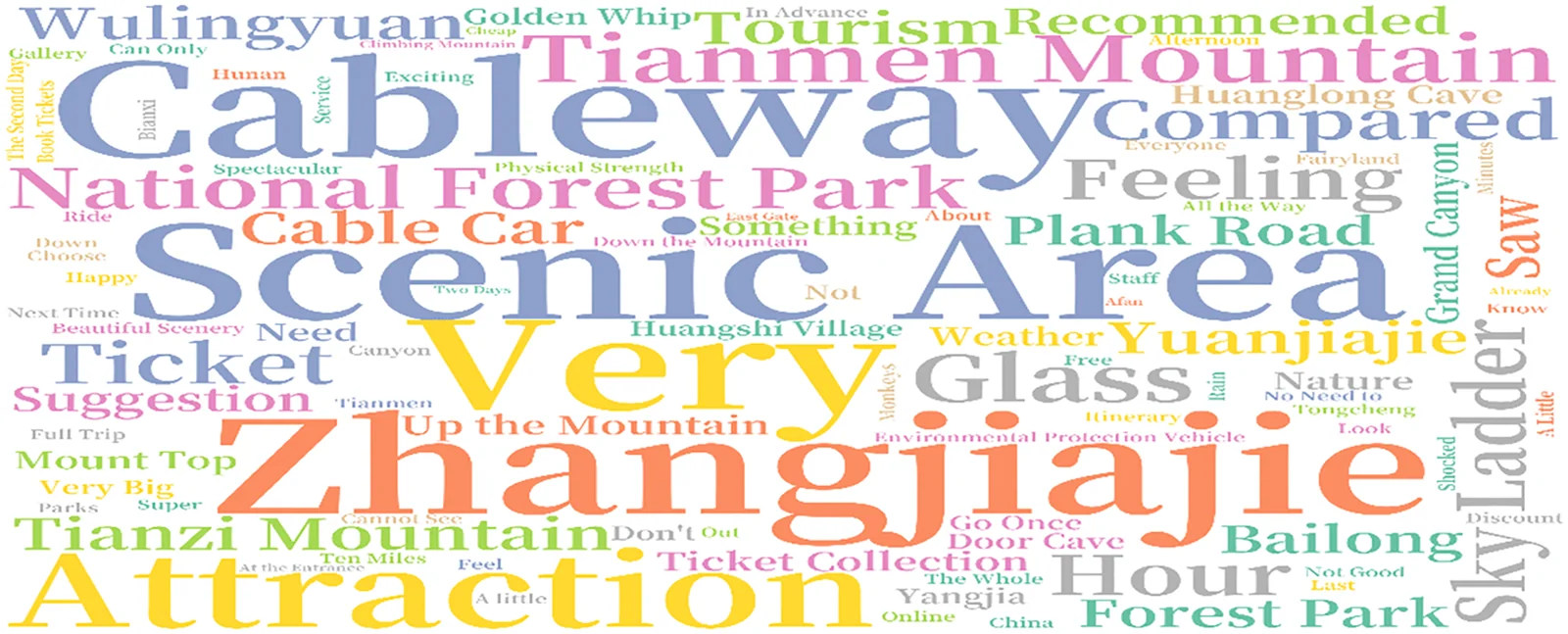
Word cloud of high-frequency keywords in Zhangjiajie tourist reviews. The visualization is generated from 8,592 user-generated reviews using Python-based text mining and word cloud analysis.

### 3.4. Topic interpretation and construct development

#### 3.4.1. Establishing the LDA topic model.

An LDA topic model was applied to the preprocessed corpus to extract latent emotional and experiential themes. The number of topics was determined using perplexity and coherence metrics to balance statistical fit and semantic interpretability [6,12,14,50]. Topic solutions ranging from 1 to 15 were evaluated, and the corresponding metric trends were examined (Figs 3 and 4). Based on the joint optimization of the two criteria, the 11-topic solution was selected as the final model configuration.

**Fig 3:**
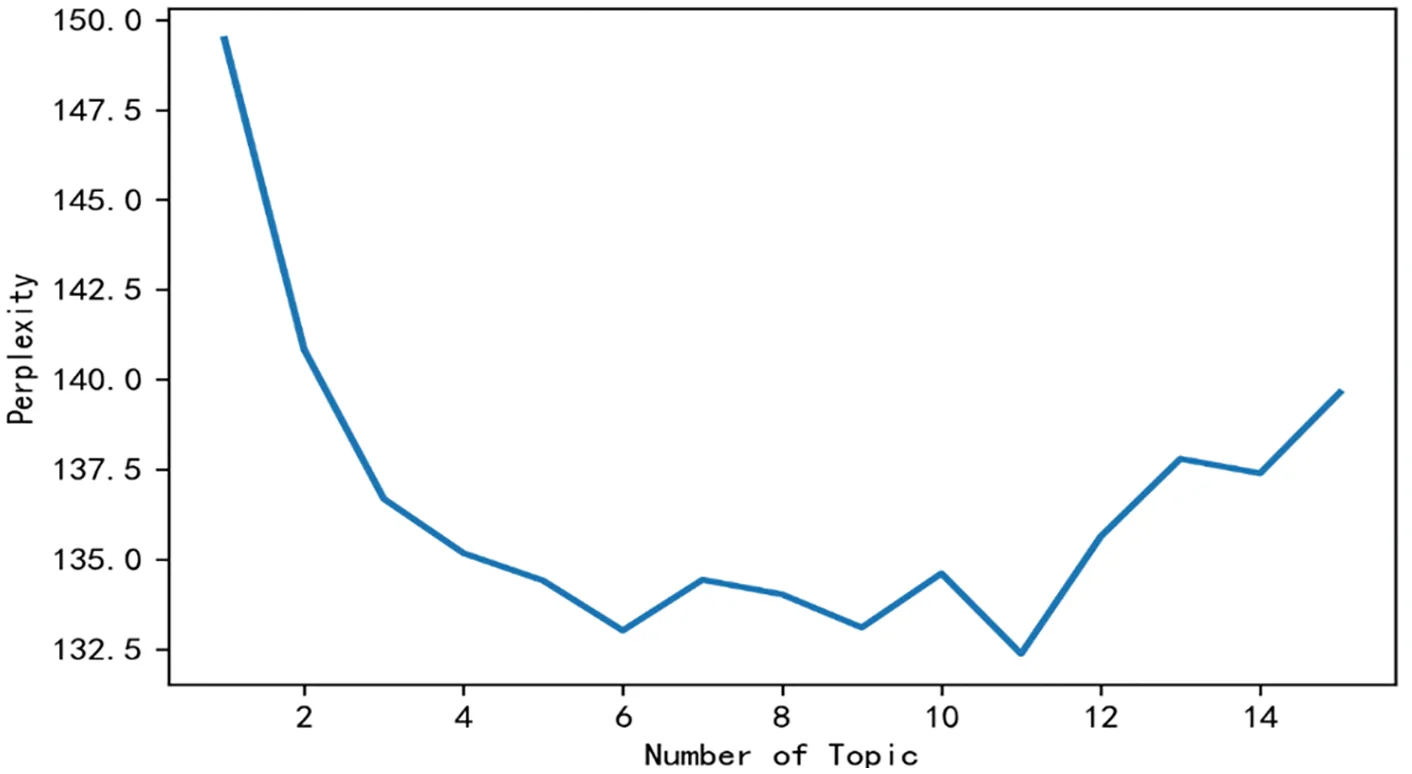
Topic perplexity scores across different topic numbers. The figure shows changes in perplexity as the number of topics varies from 1 to 15, supporting the selection of an 11-topic solution.

**Fig 4:**
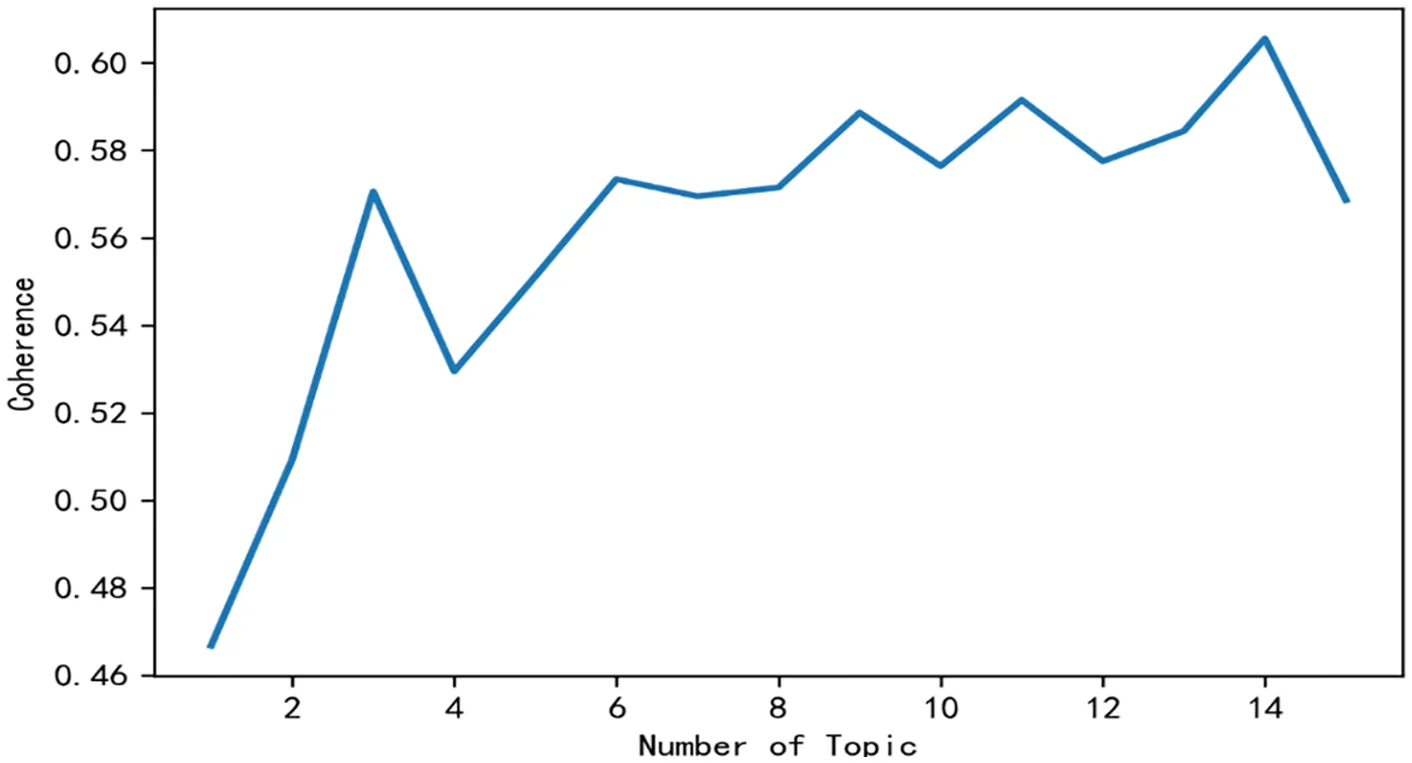
Topic coherence scores across different topic numbers. This figure illustrates the variation in coherence values for models with 1–15 topics, indicating improved semantic consistency near the selected model.

The LDA model was implemented using the Gensim library in Python. The hyperparameters were set to α = 0.1 and β = 0.01, and the model was run for 5,000 iterations. The resulting intertopic distance map, visualized using pyLDAvis (Fig. 5), shows that most topics are well separated, with limited localized overlap, supporting the interpretability of the identified thematic structure.

**Fig 5:**
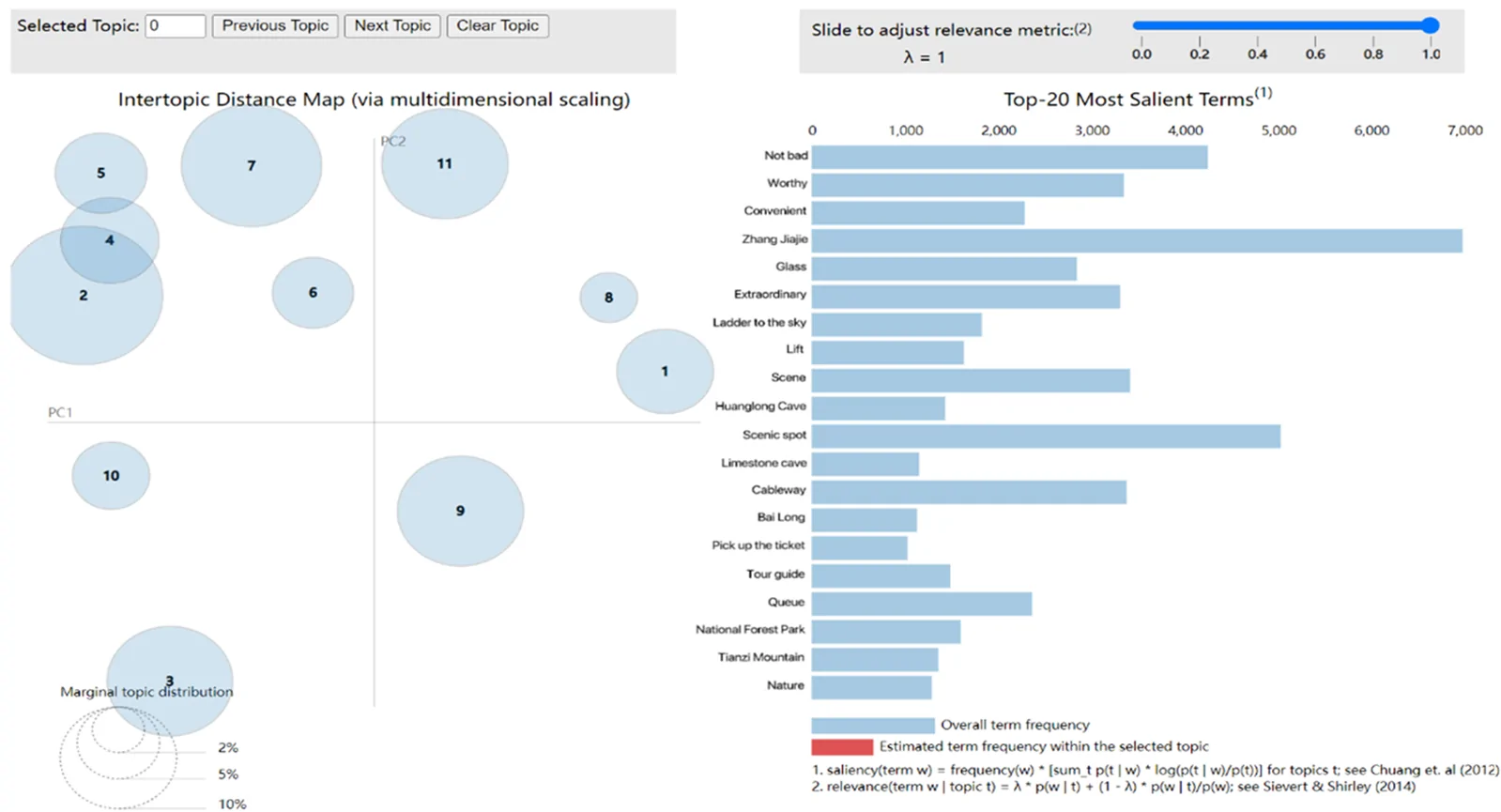
LDA topic clustering visualization using pyLDAvis. The figure illustrates the spatial distribution of 11 topics, where inter-topic distances reflect semantic similarity and structural independence.

#### 3.4.2. Topic naming and dimensional categorization.

After determining the optimal 11-topic solution, topic labels were assigned through a structured multi-source triangulation procedure combining keyword extraction, representative document sampling, literature-based interpretation, and expert evaluation. To ensure methodological transparency and reproducibility, five experts in tourism management and ecotourism independently reviewed the LDA outputs and proposed initial topic labels based on high-probability keywords and representative texts. A formal iterative consensus procedure was then conducted, involving comparison of labeling differences, semantic alignment checks, and refinement of ambiguous categories until agreement was reached among experts.

The final topics (T1–T11) were defined as follows. T1 Vegetation landscape refers to forest and ecological scenery within protected natural environments [32]. T2 Time nodes capture temporal constraints such as queuing time and seasonal conditions [59]. T3 Visitor perceptions and evaluations reflect overall satisfaction and affective judgments of travel experience [34]. T4 Scenic services involve service encounters and interaction processes within scenic areas [44]. T5 Scenic management refers to ticketing, pricing, and reservation systems [60,61]. T6 Water landscape represents karst cave environments and water-based scenic experiences [54]. T7 Architectural landscape includes artificial structures such as glass walkways and cable cars [62,63]. T8 Scenic facilities refer to infrastructure supporting accessibility and mobility [24]. T9 Scenic impressions capture holistic cognitive–affective evaluations of the destination experience [5,64]. T10 Visitor activities refer to participatory behaviors such as sightseeing and exploration [46]. T11 Mountain landscape describes peak-forest geomorphological features that define the destination’s spatial identity [29].

To ensure theoretical validity and construct consistency, the identified topics were further mapped into higher-order dimensions based on triangulation of the Resource-Based View (RBV), Service-Dominant Logic (SDL), and Experience Economy theory. Specifically, Tourist Attractions (T1, T6, T7, T11) represent destination resource endowments [32,41]. Tourist Reception (T4, T5, T8) reflects service systems and management structures [44, 65]. And Tourist Experience (T2, T3, T9, T10) captures temporal, cognitive, emotional, and behavioral components of visitor experience formation [34,46]. This theory-driven categorization ensures alignment between data-driven topic extraction and the definition of conceptual constructs.

Finally, the LDA-derived topics were operationalized as variables for the subsequent fuzzy DEMATEL and DANP analyses. As summarized in Table 2, each topic was assigned to a theoretically grounded higher-order dimension and defined in terms of its representative keywords and theoretical interpretation. This operationalization establishes methodological continuity between semantic topic extraction and causal–structural modeling, enabling a transparent and reproducible transformation of textual data into a system-level analysis of tourists’ emotional experiences.

**Table 2:** Classification of LDA-derived topics into dimensions, sub-criteria, and theoretical interpretations.

Dimensions	Sub-criteria	Topic	Representative Keywords	Theoretical Interpretation
Tourist Attractions (***TA***)	Vegetation Landscape (***VL***)	T1	Zhangjiajie, National Forest Park, World Natural Heritage, scenic spot	Forest ecology and vegetation landscapes constitute core natural resources that shape destinations’ competitive advantage [32].
	Water Landscape (***WL***)	T6	Huanglong Cave, Karst cave, boat, stalagmite	Karst and water-based environments provide restoration and aesthetic pleasure, influencing emotional experience [54].
	Mountain Landscape (***ML***)	T11	Tianzi Mountain, Yuanjiajie, Huangshizhai, peaks	Peak-forest landforms and mountain settings enhance destination image and satisfaction [29].
	Architectural Landscape (***AL***)	T7	Glass skywalk, cable car, Tianmen, sky ladder	Iconic artificial structures strengthen symbolic meaning and landscape recognizability [62,63].
Tourist Reception (***TR***)	Scenic Area Facilities (***SAF***)	T8	Elevator, Bailong, sightseeing, uphill/downhill	Functional accessibility of infrastructure directly affects visitor satisfaction and emotional responses [24].
	Scenic Area Services (***SAS***)	T4	Guide, explanation, staff, attitude	Service encounters and staff interaction represent key elements of value co-creation [44].
	Scenic Area Management (***SAM***)	T5	Tickets, admission, prices, reservations, queuing	Ticketing, pricing, and management systems exert substantial impacts on satisfaction and perceptions of fairness [60,61].
Tourist Experience (**TE**)	Time Nodes (***TN***)	T2	Queue, time, hour, weather, peak season	Temporal and climatic conditions shape mood, comfort, and satisfaction [59].
	Visitor Perception and Evaluation (***VPE***)	T3	Good, worthy, excellent, recommended, beautiful	Emotional responses and evaluative judgments influence revisit and recommendation intentions [34].
	Visitor Activities (***VA***)	T10	Sightseeing, boating, boardwalk, climbing, thrills	Participatory and interactive activities enrich experiential value and memory formation [46,66].
	Scenic Area Impressions (***SAI***)	T9	Nature, beautiful scenery, fairyland, clouds and mist, awe	Cognitive-emotional imagery forms the overall destination image [5, 64].

Notes: *TA = Tourist Attractions; TR = Tourist Reception; TE = Tourist Experience.*

*Topics (T1–T11) were identified using latent Dirichlet allocation (LDA) and categorized through expert consensus. The dimensional grouping draws on the Resource-Based View (TA), Service-Dominant Logic (TR), and Experience Economy perspectives (TE). Representative keywords are selected from the top-weight words for each topic.*

### 3.5. Fuzzy DEMATEL–DANP causal-weighting framework

This study develops a unified causal-weighting framework by integrating fuzzy DEMATEL and modified DANP to capture both causal relationships and hierarchical importance among emotional experience factors. The framework is embedded within a sequential analytical logic in which semantic extraction, causal inference, and structural prioritization are systematically connected.

#### 3.5.1. Fuzzy DEMATEL for causal structure identification.

Experts evaluated pairwise influence relationships among factors using a five-point linguistic scale, which was converted into triangular fuzzy numbers (Table 3). The CFCS (Converting Fuzzy data into Crisp Scores) method was applied to transform fuzzy assessments into crisp values [67], yielding the initial direct-relation matrix $A=\left[a_{ij}\right]$ (see S1 Appendix for computational details). The resulting matrix was then normalized to obtain the normalized direct-relation matrix X, which serves as the basis for causal analysis within the DEMATEL framework.

**Table 3:** Linguistic variables and corresponding triangular fuzzy numbers.

Linguistic variable	Triangular fuzzy number
No influence (*0*)	(0, 0, 0.25)
Very low influence (*1*)	(0, 0.25, 0.5)
Low influence (*2*)	(0.25, 0.5, 0.75)
High influence (*3*)	(0.5, 0.75, 1.0)
Very high influence (*4*)	(0.75, 1.0, 1.0)

*Note: This table presents the mapping between linguistic terms used in the DEMATEL scale and their associated triangular fuzzy numbers.*

The total influence matrix, capturing both direct and indirect effects among factors, was subsequently derived using the DEMATEL infinite-series formulation T.


$$\begin{array}{c} T\text{=}{\left[t_{ij}\right]}_{n\times n}=X(I-X{)}^{-1} \end{array}$$

Based on the total influence matrix T, the Influence Network Relation Map (INRM) was constructed to represent the system-level causal structure among emotional factors. A threshold value α, defined as the mean of all elements in the total influence matrix, was used to eliminate weak relationships and retain significant causal links.

Finally, the prominence (R+K) and relation (R−K) indicators were computed, where D and R denote the row and column sums of the total influence matrix, respectively. These indicators represent the overall influence and net causal effect of each factor within the system, allowing the identification of cause–and–effect groupings in the emotional experience structure.

#### 3.5.2 Modified DANP for hierarchical weighting.

A modified DANP approach was adopted to capture hierarchical dependencies among emotional dimensions. In the traditional DANP framework, the supermatrix is constructed directly from the total influence matrix, implicitly assuming equal importance across clusters. However, emotional experience systems typically exhibit hierarchical structures with heterogeneous inter-cluster importance. To address this limitation, dimension-level weights derived from the DEMATEL total influence matrix were incorporated into the ANP supermatrix construction, enabling inter-cluster relationships to be adjusted according to hierarchical differences while preserving the causal structure identified by DEMATEL [23,31]. This modification follows the established DEMATEL–ANP integration framework widely applied in complex system decision analysis [23,24].

The weighted supermatrix was iteratively multiplied until convergence, yielding the limiting supermatrix.:


$$\begin{array}{c} (W_{c}{)}^{\infty }=\underset{k\to \infty }{lim}{\left(W_{D}\cdot W_{c}^{un}\right)}^{k} \end{array}$$

The resulting limiting weights reflect both the strength of causal influence and the hierarchical importance of emotional factors, providing a consistent extension of the standard DANP framework [23]. Computational details are provided in S2 Appendix.

#### 3.5.3 Model integration.

The proposed framework establishes a closed-loop causal–hierarchical system that integrates semantic extraction, causal analysis, and structural weighting into a unified modeling process. LDA is first employed to identify latent emotional themes from user-generated content, providing the semantic foundation for subsequent analysis. These themes are then transformed into a causal network through fuzzy DEMATEL, which captures directional relationships among emotional dimensions. Finally, the modified DANP approach translates the causal structure into hierarchical weights that reflect both direct and indirect dependencies among factors. This sequential transformation ensures methodological consistency across all analytical stages and establishes an integrated modeling logic in which semantic representation (LDA), causal structure (DEMATEL), and hierarchical prioritization (DANP) are systematically aligned to explain the formation of emotional experiences in tourism.

## 4. Results

### 4.1. Expert participation and questionnaire design

In fuzzy DEMATEL–DANP analysis, expert judgment is the primary input for constructing the direct-influence matrix, and methodological validity depends more on the quality and consistency of expertise than on sample size. Accordingly, expert panel design is critical to ensuring analytical robustness. Prior methodological studies suggest that relatively small but well-qualified panels are sufficient to yield stable decision outcomes, with around 10 experts considered adequate to capture diverse perspectives without introducing excessive judgmental noise [68]. In addition, excessively large expert groups may reduce consistency without improving analytical accuracy [69]. Based on these considerations and to balance rigor with feasibility, this study recruited a panel of seventeen experts.

The panel consisted of five senior scenic area managers, five tourism enterprise executives, and seven academic scholars with over ten years of experience in tourism management research. This multidisciplinary composition integrates operational knowledge, managerial practice, and academic expertise, thereby enhancing the reliability and contextual relevance of expert evaluations in destination management contexts. Experts were recruited through correspondence questionnaires between 1 November and 15 December 2024. Participation was voluntary, and implied informed consent was obtained through completion and return of the questionnaire. No minors participated. No identifying or sensitive information was included in the analytical dataset, and all responses were anonymized, aggregated at the group level, and used exclusively for academic research.

### 4.2. INRM analysis based on fuzzy DEMATEL

Fuzzy DEMATEL was applied to construct the structural relationships among emotional dimensions and sub-criteria derived from LDA. The initial direct-influence matrices were obtained after transforming expert linguistic evaluations into triangular fuzzy numbers and defuzzifying them into crisp values (Tables 4 and 5). Based on normalization and matrix convergence procedures, the total influence matrices were derived (Tables 6 and 7), and the Influence Network Relation Maps (INRMs) were constructed by retaining influence values above the mean threshold (Figs 6 and 7).

**Table 4:** Initial fuzzy direct-influence matrix of dimensions.

$A_{D}$	*TA*	*TR*	*TE*
**Tourist Attractions (*TA*)**	0.000	3.063	2.512
**Tourist Reception (*TR*)**	2.783	0.000	2.556
**Tourist Experience (*TE*)**	3.063	0.889	0.000

**Table 5:** Initial fuzzy direct-influence matrix of sub-criteria.

$A_{c}$	*VL*	*WL*	*ML*	*AL*	*SAF*	*SAS*	*SAM*	*TN*	*VPE*	*VA*	*SAI*
***VL***	0.000	2.563	2.745	2.663	2.443	1.743	2.236	2.773	2.063	2.034	2.125
***WL***	2.542	0.000	1.665	1.937	2.783	2.125	2.783	1.889	2.632	2.443	2.125
***ML***	1.674	0.532	0.000	2.432	2.034	2.934	2.322	2.063	2.443	2.632	2.783
***AL***	1.833	1.233	2.125	0.000	2.114	1.422	1.982	1.938	2.125	2.443	2.553
***SAF***	2.332	1.164	2.783	1.223	0.000	1.335	1.833	1.233	2.742	2.568	2.784
***SAS***	1.742	1.833	2.834	2.063	2.783	0.000	2.542	1.335	2.446	2.125	2.063
***SAM***	1.833	2.889	1.743	1.889	2.063	2.674	0.000	2.843	2.243	2.326	2.432
***TN***	1.643	1.982	1.165	1.335	2.934	2.063	2.632	0.000	2.543	2.332	2.125
***VPE***	1.982	1.243	1.674	1.883	2.332	2.544	2.063	1.556	0.000	1.832	2.543
***VA***	1.742	1.223	1.422	1.675	2.544	2.634	2.245	1.742	2.125	0.000	1.982
***SAI***	1.543	1.742	1.335	1.563	2.633	2.118	2.443	1.834	2.332	2.063	0.000

**Table 6:** Total influence matrix with centrality and causality (dimensions).

$T_{D}$	*TA*	*TR*	*TE*	R	R+K	R−K	Group
***TA***	2.033	1.914	2.140	6.087	12.348	−0.174	Affected
***TR***	2.291	1.517	2.085	5.893	10.711	1.075	Cause
***TE***	1.937	1.386	1.438	4.762	10.425	−0.902	Affected
K	6.261	4.818	5.664				

*Note: The threshold value α = 1.860. Values greater than the threshold are shown in bold.*

**Table 7:** Total influence matrix with centrality and causality (sub-criteria).

$T_{c}$	*VL*	*WL*	*ML*	*AL*	*SAF*	*SAS*	*SAM*	*TN*	*VPE*	*VA*	*SAI*	R	R+K	R−K	*Group*
***VL***	0.451	0.490	**0.566**	**0.541**	**0.667**	**0.579**	**0.624**	**0.558**	**0.635**	**0.614**	**0.635**	6.3617	11.561	1.163	Cause
***WL***	**0.537**	0.390	**0.522**	0.508	**0.668**	**0.583**	**0.633**	0.519	**0.644**	**0.617**	**0.625**	6.2465	10.816	1.677	Cause
***ML***	0.481	0.391	0.434	0.502	**0.613**	**0.586**	**0.589**	0.500	**0.608**	**0.595**	**0.619**	5.9178	11.298	0.537	Cause
***AL***	0.451	0.384	0.475	0.376	**0.569**	0.492	**0.534**	0.460	**0.553**	**0.546**	**0.567**	5.4066	10.521	0.293	Cause
***SAF***	0.474	0.385	0.503	0.430	0.496	0.496	**0.534**	0.441	**0.580**	**0.556**	**0.581**	5.4753	12.136	−1.185	Affected
***SAS***	0.487	0.437	**0.541**	0.491	**0.640**	0.481	**0.598**	0.477	**0.611**	**0.581**	**0.597**	5.9421	11.866	0.018	Cause
***SAM***	0.512	0.496	0.523	0.506	**0.646**	0.603	**0.533**	**0.551**	**0.632**	**0.613**	**0.634**	6.2477	12.491	0.004	Cause
***TN***	0.466	0.429	0.462	0.446	**0.623**	**0.536**	**0.579**	0.406	**0.592**	**0.565**	**0.575**	5.6786	10.937	0.420	Cause
***VPE***	0.456	0.385	0.461	0.447	**0.576**	**0.529**	**0.536**	0.446	0.473	**0.524**	**0.565**	5.3996	11.834	−1.035	Affected
***VA***	0.443	0.380	0.446	0.434	**0.576**	**0.526**	**0.535**	0.446	**0.546**	0.448	**0.539**	5.3189	11.509	−0.872	Affected
***SAI***	0.441	0.402	0.447	0.434	**0.586**	0.514	**0.548**	0.454	**0.559**	**0.531**	0.471	5.3886	11.797	−1.020	Affected
K	5.199	4.570	5.381	5.114	6.6602	5.9241	6.2437	5.2584	6.4343	6.1906	6.4087				

Note: The threshold value α = 0.524. Values greater than the threshold are shown in bold.

**Fig 6:**
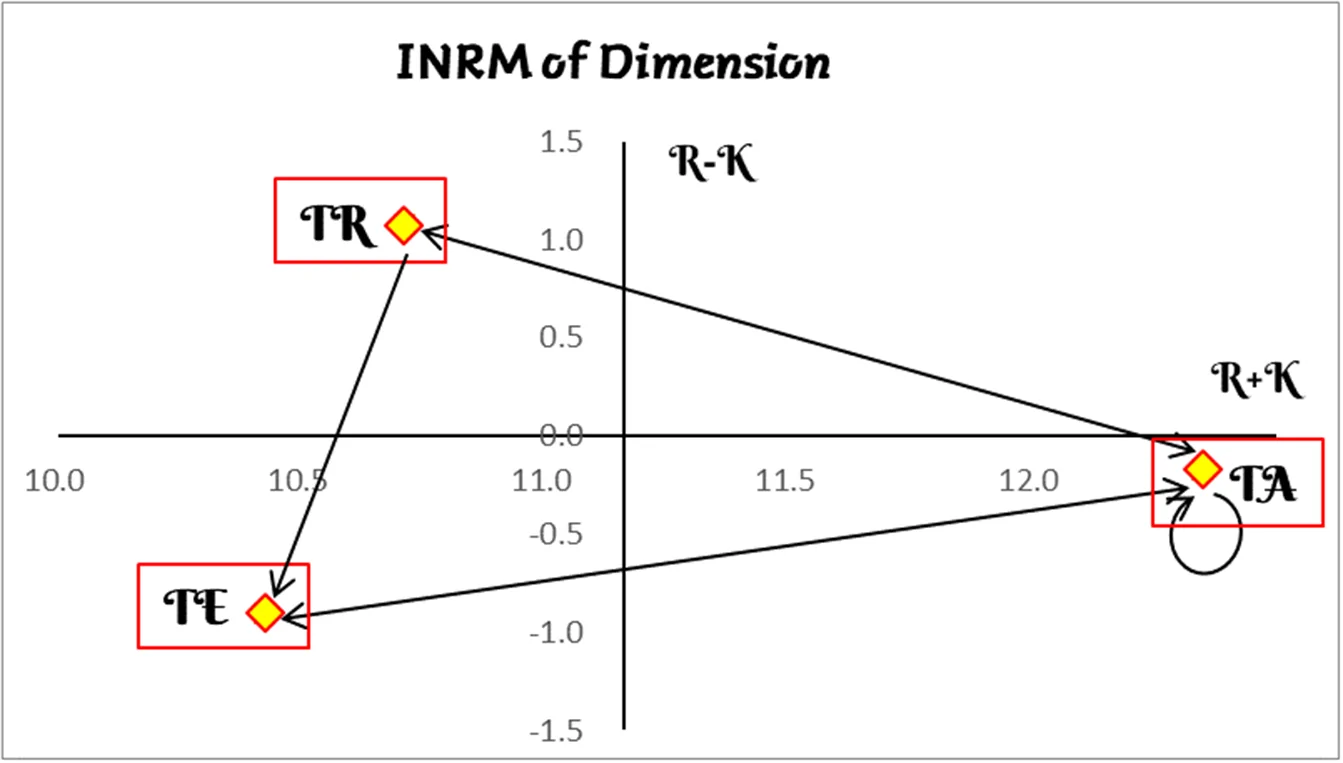
Influence Network Relationship Map (INRM) of dimensions.

**Fig 7:**
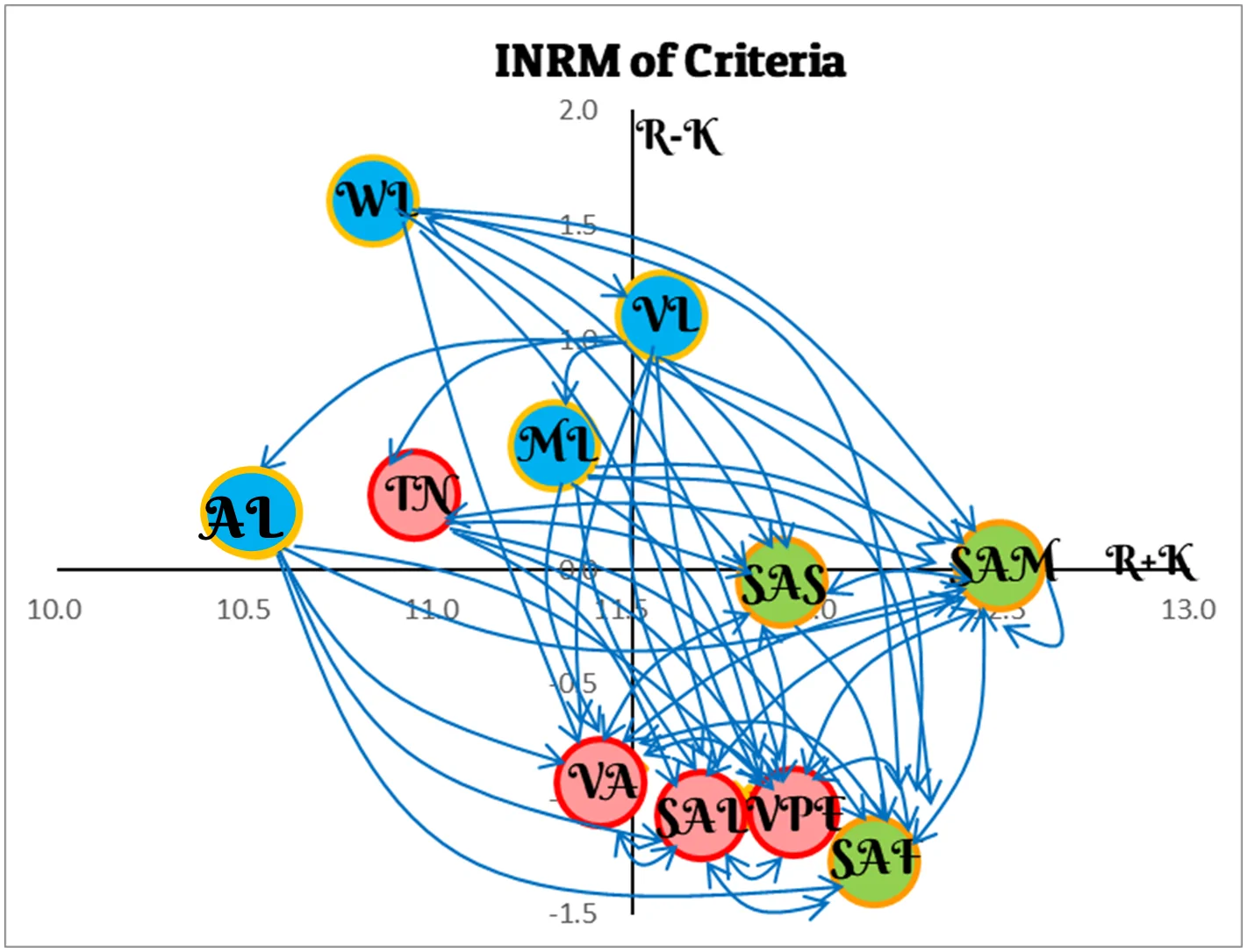
Influence Network Relationship Map (INRM) of sub-criteria.

(1) Dimension-level INRM

As shown in Table 6 and Fig. 6, the three dimensions exhibit strong interdependencies. ***TA*** demonstrates the highest centrality, followed by ***TR*** and ***TE***, indicating that attraction-related factors are more structurally embedded within the emotional experience system. In terms of causality, TR is identified as the only causal dimension (positive value), while TA and TE function as net receivers (negative values), suggesting that reception-related factors act as the primary driving force within the system. Recent studies similarly indicate that improvements in service and management capacity enhance both attraction and experience subsystems within destination systems [30,44], reinforcing the critical role of the reception dimension as a structural leverage point in shaping overall system performance.

(2) Sub-criteria-level Analysis

Table 7 and Fig 7 reveal a densely interconnected causal structure among the eleven indicators. Indicators with the highest centrality values—***SAM***, ***SAF***, ***SAS***, ***VA***, and ***SAI***—function as core nodes within the emotional experience network, indicating their structural importance in maintaining system connectivity. In terms of causality, ***VL***, ***WL***, ***ML***, ***SAM***, ***SAS***, and ***TN*** exhibit positive influence values and are identified as causal sub-criteria, suggesting that environmental quality, management systems, and temporal arrangements jointly act as upstream drivers shaping downstream emotional responses and overall destination evaluation. This causal configuration is consistent with prior studies highlighting that coordinated destination management and environmental design constitute the structural foundation of tourism experience systems, ultimately influencing tourists’ emotional outcomes and satisfaction [44,46].

### 4.3. Path relationship analysis

Based on Table 7 and Fig 7, this study identified several pathways of influence among the eleven variables. To clarify how these pathways operate, this section uses ***AL*** as an example to demonstrate how fuzzy DEMATEL helps identify structural causal paths.

- ***AL*** → ***SAF***, ***SAM***, ***VPE***, ***VA*** and ***SAI***.

***AL*** shapes tourists’ emotional experiences primarily through the scenic area’s spatial layout and visual imagery. Keywords such as “boardwalk,” “park,” and “mountain temple” reflect its aesthetic and cultural characteristics. These spatial elements determine main tour routes and viewing points, which in turn influence the configuration of facilities and the design of the tour guidance system. Infrastructure components such as “cableway,” “elevator,” and “eco-friendly vehicle” need to align with the landscape structure to ensure accessibility, smooth visitor flow, and safety [30,63].

The uniqueness and accessibility of ***AL*** also influence the tourist reception subsystem, affecting staffing, route planning, queue arrangements, and visitor dispatching [16,44]. These operational mechanisms shape perceived service efficiency and management quality, indicating that ***AL***’s influence on ***SAF*** and ***SAM*** is transmitted through coordinated operational processes and subsequently extends to ***VPE***, ***VA***, and ***SAI***. At the experiential level, architectural landscapes shape tourists’ movement patterns, visual attention, and on-site interactions, with activities such as photography, sightseeing, hiking, and queuing embedded within spatial configurations, thereby reinforcing ***AL***’s influence on ***VA***. In addition, the visual and cultural symbolism of architectural landscapes generates strong emotional and aesthetic responses, as reflected in frequent descriptors such as “spectacular,” “shocking,” “recommended,” and “gallery,” which are closely associated with evaluative perceptions and destination impressions [34,46].

Overall, ***AL*** influences ***SAF***, ***SAM***, ***VPE***, ***VA***, and ***SAI*** through three interconnected pathways—spatial organization, aesthetic symbolism, and service-synergetic effects—highlighting its structural role within the emotional experience system. This finding is consistent with studies emphasizing the central role of cultural landscapes and environmental cues in shaping tourists’ emotions and experiential outcomes [17,34,46].

As mentioned earlier, *fuzzy DEMATEL enables the identification of structural causal pathways under limited-sample conditions, making it particularly suitable for exploratory causal analysis compared with covariance-based methods such as AMOS and SmartPLS, which require larger observational datasets.* The resulting causal network provides a stable structural basis for subsequent DANP weighting analysis. Although ***AL*** is used as an illustrative example, other variables in the system exhibit similar patterns of causal propagation.

### 4.4. Weight calculation based on DANP

Building on the INRM results, the modified DANP approach was applied to derive the global weights of dimensions and sub-criteria in the Zhangjiajie emotional experience system [31,70]. The dimension-level influence matrix was normalized and iteratively processed until convergence, yielding a stable limit matrix that reflects inter-dimensional dependencies and hierarchical influence structures. At the criteria level, the total influence matrix was transformed into an unweighted supermatrix, then adjusted with dimension-level weights to obtain the weighted supermatrix. The final limit supermatrix was obtained via repeated multiplication until convergence (see Table 8, Table 9, Table 10 and S1 Appendix), yielding the global priority rankings for all emotional factors in Table 11.

**Table 10:** Limit supermatrix of dimensions and sub-criteria.

Dimension	*TA*	*TR*	*TE*	Criteria	*VL*	*WL*	*ML*	*AL*	*SAF*	*SAS*	*SAM*	*TN*	*VPE*	*VA*	*SAI*
***TA***	0.374	0.374	0.374	***VL***	0.096	0.096	0.096	0.096	0.096	0.096	0.096	0.096	0.096	0.096	0.096
***TR***	0.290	0.290	0.290	***WL***	0.084	0.084	0.084	0.084	0.084	0.084	0.084	0.084	0.084	0.084	0.084
***TE***	0.336	0.336	0.336	***ML***	0.099	0.099	0.099	0.099	0.099	0.099	0.099	0.099	0.099	0.099	0.099
				***AL***	0.094	0.094	0.094	0.094	0.094	0.094	0.094	0.094	0.094	0.094	0.094
				***SAF***	0.102	0.102	0.102	0.102	0.102	0.102	0.102	0.102	0.102	0.102	0.102
				***SAS***	0.091	0.091	0.091	0.091	0.091	0.091	0.091	0.091	0.091	0.091	0.091
				***SAM***	0.096	0.096	0.096	0.096	0.096	0.096	0.096	0.096	0.096	0.096	0.096
				***TN***	0.073	0.073	0.073	0.073	0.073	0.073	0.073	0.073	0.073	0.073	0.073
				***VPE***	0.089	0.089	0.089	0.089	0.089	0.089	0.089	0.089	0.089	0.089	0.089
				***VA***	0.086	0.086	0.086	0.086	0.086	0.086	0.086	0.086	0.086	0.086	0.086
				***SAI***	0.089	0.089	0.089	0.089	0.089	0.089	0.089	0.089	0.089	0.089	0.089

**Table 11:** Weights of dimensions and sub-criteria.

Dimension	Criteria	Weighting	Ranking
**Tourist Attractions (TA)**		**0.374**	
	**Vegetation landscape (*VL*)**	0.096	3
	**Water landscape (*WL*)**	0.084	10
	**Mountain landscape (*ML*)**	0.099	2
	**Architectural landscape (*AL*)**	0.094	5
**Tourist Reception (TR)**		**0.290**	
	**Scenic area facilities (*SAF*)**	0.102	1
	**Scenic area services (*SAS*)**	0.091	6
	**Scenic area management (*SAM*)**	0.096	3
**Tourist Experience (TE)**		**0.336**	
	**Time nodes (*TN*)**	0.073	11
	**Visitor perception and evaluation (*VPE*)**	0.089	7
	**Visitor activities (*VA*)**	0.086	9
	**Scenic area impressions (*SAI*)**	0.089	7

At the dimension level, ***TA*** exhibits the highest weight (0.374), followed by ***TE*** (0.336) and ***TR*** (0.290). This indicates that although service processes and experiential interactions are important, natural and cultural resources remain the primary structural drivers of tourists’ emotional experiences.

**Table 8:** Unweighted sumatbrix of sub-criteria.

Criteria	*VL*	*WL*	*ML*	*AL*	*SAF*	*SAS*	*SAM*	*TN*	*VPE*	*VA*	*SAI*
***VL***	0.220	0.274	0.266	0.268	0.264	0.249	0.251	0.258	0.261	0.260	0.256
***WL***	0.239	0.199	0.217	0.228	0.215	0.224	0.243	0.238	0.220	0.223	0.233
***ML***	0.277	0.267	0.240	0.282	0.281	0.276	0.257	0.256	0.263	0.262	0.259
***AL***	0.264	0.259	0.277	0.223	0.240	0.251	0.248	0.247	0.255	0.255	0.252
***SAF***	0.357	0.355	0.343	0.357	0.325	0.372	0.363	0.358	0.351	0.352	0.355
***SAS***	0.310	0.310	0.328	0.308	0.325	0.280	0.338	0.308	0.322	0.321	0.312
***SAM***	0.334	0.336	0.329	0.335	0.350	0.348	0.299	0.333	0.327	0.327	0.333
***TN***	0.228	0.216	0.215	0.216	0.204	0.211	0.227	0.190	0.222	0.225	0.225
***VPE***	0.260	0.268	0.262	0.260	0.269	0.270	0.260	0.277	0.236	0.276	0.277
***VA***	0.251	0.256	0.256	0.257	0.258	0.256	0.252	0.264	0.261	0.227	0.264
***SAI***	0.260	0.260	0.266	0.267	0.269	0.264	0.261	0.269	0.281	0.272	0.234

**Table 9:** Weighted supermatrix of dimensions and sub-criteria.

Dimension	*TA*	*TR*	*TE*	Criteria	*VL*	*WL*	*ML*	*AL*	*SAF*	*SAS*	*SAM*	*TN*	*VPE*	*VA*	*SAI*
***TA***	0.334	0.389	0.407	***VL***	0.074	0.092	0.089	0.089	0.103	0.097	0.098	0.105	0.106	0.106	0.104
***TR***	0.314	0.257	0.291	***WL***	0.080	0.067	0.072	0.076	0.084	0.087	0.095	0.097	0.090	0.091	0.095
***TE***	0.352	0.354	0.302	***ML***	0.092	0.089	0.080	0.094	0.109	0.107	0.100	0.104	0.107	0.107	0.106
				***AL***	0.088	0.087	0.093	0.074	0.093	0.098	0.096	0.101	0.104	0.104	0.102
				***SAF***	0.112	0.112	0.108	0.112	0.084	0.096	0.093	0.104	0.102	0.102	0.103
				***SAS***	0.097	0.097	0.103	0.097	0.084	0.072	0.087	0.090	0.094	0.093	0.091
				***SAM***	0.105	0.106	0.104	0.105	0.090	0.090	0.077	0.097	0.095	0.095	0.097
				***TN***	0.080	0.076	0.076	0.076	0.072	0.074	0.080	0.057	0.067	0.068	0.068
				***VPE***	0.091	0.094	0.092	0.091	0.095	0.095	0.092	0.084	0.071	0.083	0.084
				***VA***	0.088	0.090	0.090	0.090	0.091	0.091	0.089	0.080	0.079	0.068	0.080
				***SAI***	0.091	0.091	0.094	0.094	0.095	0.093	0.092	0.081	0.085	0.082	0.071

At the sub-criteria level, ***SAF*** (0.102) emerges as the most influential indicator, followed by ***ML*** (0.099), ***VL*** (0.096), ***SAM*** (0.096), and ***AL*** (0.094). These high-weight indicators function as primary drivers within the emotional experience system, highlighting the importance of facility completeness, management effectiveness, and the integration of natural and built landscapes in shaping emotional outcomes [32,44]. Notably, these indicators fall within the causal quadrant of the INRM, suggesting that the “management–facilities–landscape” nexus underpins the structural configuration of the emotional experience system. This pattern is consistent with the resource-based view, which emphasizes the role of core environmental and managerial resources in destination competitiveness [71], and aligns with studies that highlight the importance of cultural landscapes in the formation of experiential value [45]. The combined influence of these resources enhances tourists’ perceived value and emotional outcomes as documented in tourism experience research [8,34].

In contrast, ***VPE*** (0.089) and ***SAI*** (0.089) serve as outcome-oriented indicators that reflect post-experience evaluations, whereas ***TN*** (0.073) exhibits the lowest weight, indicating relatively limited sensitivity to temporal constraints in shaping the overall emotional experience [7,46,59].

In summary, the DANP results confirm a three-layer structure of “tourist attractions–reception–experience,” where ***TR*** functions as the main driving layer, ***TA*** as the resource foundation layer, and ***TE*** as the experiential outcome layer. The derived weights provide a quantitative basis for subsequent quadrant analysis and managerial implications.

## 5. Discussion

This study develops a four-quadrant emotional experience system for Zhangjiajie using fuzzy DEMATEL (Fig 7), showing that tourist emotional formation is a structured system rather than isolated experiential attributes, emerging through interactions among inputs, contextual conditions, behavioral transformation, and outcomes.

(1) Quadrant I: High Causality and High Centrality

This quadrant includes ***SAM***, ***SAS***, and ***VL***, forming the system’s input layer, which comprises primary environmental and service-driven stimuli. ***SAM*** reduces crowding and negative affect through staffing, safety supervision, and emergency response, enhancing perceived control and safety [71,72]. ***SAS*** reflects Service-Dominant Logic (SDL), in which interpretive, guiding, and interpersonal services reduce uncertainty and strengthen psychological security [8,44]. ***VL*** provides ecological immersion and sensory stimulation that facilitate attentional restoration and emotional recovery, strengthening place attachment [34,46,54]. These stimuli operate through cognitive appraisal, in which environmental and service cues are interpreted as signals of safety, quality, and meaning, thereby generating trust and comfort [7,33,38], reflecting the joint operation of the Resource-Based View (RBV) and SDL in structuring destination value creation [30,45].

(2) Quadrant II: High Causality and Low Centrality

This quadrant includes ***WL***, ***ML***, ***AL***, and ***TN***, which constitute the contextual conditioning layer that shapes spatial–temporal experience. ***WL*** and ***ML*** enhance sensory immersion and perceived natural quality [32]. ***AL*** structures spatial cognition and movement orientation through cultural symbolism embedded in landscape design [2,73]. ***TN*** regulates temporal order through reservation systems, queue management, and visitor-flow control, improving perceived predictability and environmental stability [59,72]. These factors do not directly generate emotional responses but shape the conditions for cognitive appraisal by structuring attention allocation and environmental interpretation, thereby providing contextual constraints for psychological processing within the overall experience system [30].

(3) Quadrant III: Low Causality and Low Centrality.

This quadrant includes ***VA***, acting as the behavioral transformation layer within the system. ***VA*** converts environmental stimuli into embodied participation, enhancing immersion and engagement [7,46]. From a psychological perspective, this operates through attention–engagement mechanisms that strengthen cognitive absorption and affective resonance during the formation of experience [12,48], thereby positioning VA as a conversion interface between structural conditions and experiential outcomes. Importantly, behavioral engagement also feeds back into the system, reinforcing upstream environmental and perceptual conditions and enabling adaptive system adjustment [44,46].

(4) Quadrant IV: Low Causality and High Centrality.

This quadrant includes ***SAF***, ***VPE***, and ***SAI***, representing the outcome layer of the tourism emotional experience system. ***SAF*** directly shapes tourists’ immediate emotional responses and behavioral tendencies, with improvements in facility convenience and service efficiency significantly enhancing overall satisfaction [2]. ***VPE*** captures expectation–perception alignment and provides evaluative feedback for system optimization [33,34,38,64]. ***SAI*** integrates cognitive and emotional responses, forming destination memory and influencing revisit intention and word-of-mouth behavior [7,30]. These outcomes serve as feedback signals that adjust the upstream structural, contextual, and behavioral layers, forming a closed-loop emotional experience system [44,54] and reflecting an affective integration process in which cognitive appraisal outcomes are consolidated into stable judgments and behavioral dispositions [12,48].

Overall, the four-quadrant structure reveals a hierarchical emotional system in which tourism emotion emerges from structured interactions among environmental stimuli, contextual conditions, behavioral transformation, and experiential outcomes. The findings integrate the Resource-Based View (RBV), Service-Dominant Logic (SDL), and cognitive appraisal theory into a unified explanatory framework while clarifying their boundary condition that emotional value creation is not directly generated by resources or service interactions but is conditionally activated through cognitive-affective transformation processes, indicating that tourism emotion is a system-embedded psychological phenomenon rather than a linear outcome of structural or interactional factors.

## 6. Conclusion

### 6.1. Theoretical contributions

This study advances tourism emotion research by shifting analytical focus from static, descriptive UGC interpretation to a dynamic, system-structured causal modeling perspective, enabling the identification of hierarchical dependencies and interrelationships among experiential factors [12,28,30,49,58]. It demonstrates that emotional experience is not an isolated perceptual outcome but a system-embedded, causally interdependent process shaped by multilevel structures and psychological mechanisms [6,30,34,48].

In contrast to prior studies that treat emotional constructs as independent or directly observable outcomes, the present study reveals that emotional experience is structurally mediated rather than attribute-driven. Emotional responses emerge through cognitive appraisal processes that mediate the effects of environmental and service stimuli, extending the explanation of emotion formation from outcome-based interpretation to process-based explanation [7,36,37]. This positions emotional experience as a relational-system outcome embedded within contextual and psychological conditions rather than as a fragmented perceptual construct [6,34,48].

More importantly, the study establishes clear boundary conditions for the Resource-Based View (RBV) and Service-Dominant Logic (SDL), demonstrating that neither structural resources nor service interactions independently generate emotional value. Instead, their effects are activated through psychological transformation processes that link environmental stimuli to emotional and behavioral outcomes [8,41,45,71]. Accordingly, RBV and SDL function as structural antecedents, while cognitive appraisal operates as the core mediating mechanism within a hierarchical “resource–interaction–psychological processing–behavioral outcome” system [7,33,38,64,73].

### 6.2. Managerial implications

The causal structure and weighting results provide several managerial implications for Zhangjiajie and similar nature-based destinations. First, priority should be given to strengthening high-impact drivers such as scenic area management, service quality, and ecological maintenance, as these factors shape tourists’ cognitive appraisal and emotional responses, thereby influencing satisfaction and behavioral intentions [34,71,72]. This highlights the limitation of traditional resource-driven management logic, which assumes that natural attractions directly generate value, whereas the findings indicate that emotional experience is mediated by psychological processing of service and environmental stimuli [36,37].

Second, destination management should enhance coordination among ecological protection, cultural landscape design, and temporal regulation, particularly through reservation systems and visitor-flow management. These measures reduce uncertainty, improve perceived fairness, and stabilize emotional responses during tourism consumption, consistent with consumer psychology perspectives on cognitive control and emotional regulation in service environments [7,59]. This further extends Service-Dominant Logic (SDL), suggesting that value co-creation is not only interaction-based but also conditioned by system-level environmental design that shapes psychological experience [8].

Finally, increasing opportunities for behavioral engagement—such as thematic tours, nighttime experiences, and cultural participation—can enhance emotional immersion and experiential memory, thereby strengthening word-of-mouth intention and online sharing behavior [46,48]. From a value co-creation perspective, such engagement facilitates meaning construction and destination attachment, while improvements in facility quality and feedback systems reinforce the feedback loop between satisfaction, destination image, and loyalty [17,33,38,73].

### 6.3. Limitations and future research

Despite its contributions, this study has several limitations. First, reliance on UGC data may introduce temporal and self-selection biases, underscoring the need to integrate multimodal, real-time behavioral data to enhance the robustness of emotion measurement [12,48]. Second, the expert-driven fuzzy DEMATEL–DANP approach may involve subjective judgment, underscoring the importance of incorporating data-driven causal inference methods, such as machine learning and structural causal models, to enhance methodological robustness [30,58]. Third, the single-destination design may limit external validity, underscoring the need for cross-cultural and multi-destination comparisons to test the boundary conditions of the proposed emotional experience system [6,34].

### 6.4. Summary

By integrating large-scale UGC analysis with causal multi-criteria decision-making methods, this study identifies the hierarchical structure of tourists’ emotional experiences in Zhangjiajie. The results show that management systems, service processes, and landscape resources jointly serve as primary drivers of emotional formation, while evaluative judgments and image-related perceptions constitute outcome-level components that shape satisfaction and behavioral intentions [33,46,71]. This study develops a system-level framework that links semantic topic extraction to causal structural modeling, demonstrating that tourist emotions emerge from an interconnected rather than isolated process and providing a replicable analytical approach for emotion-oriented destination management in nature-based tourism contexts.

## Supporting information

S1 Appendix Fuzzy DEMATEL procedure. (DOCX)

S2 Appendix Modified DANP procedure. (DOCX)

S1 File PLOSOne_Human_Subjects_Research_Checklist 20260125. (DOCX)

S2 File Zhangjiajie_UGC_LDA_DEMATEL_Data. (XLSX)
