# Supplementary material for: Research on the emotional experience of online tourists in Zhangjiajie based on text mining and LDA-FDEMATEL
Source: PLoS One. 2026 Oct 1;21(10):e0355471. doi: 10.1371/journal.pone.0355471 (PMC13630242; doi:10.1371/journal.pone.0355471)
Supplement: Supplementary file 1 [file pone.0355471.s001.docx]

**Appendix A. Fuzzy DEMATEL Procedure**

Let $\tilde{h}_{ij}^{\text{(}\text{k}\text{)}}=(\text{h}_{1ij}^{\text{(}\text{k}\text{)}},\text{h}_{2ij}^{\text{(}\text{k}\text{)}},\text{h}_{3ij}^{\text{(}\text{k}\text{)}})$denote the triangular fuzzy evaluation provided by expert *k*, where $\text{h}_{1ij}^{\text{(}\text{k}\text{)}},\text{h}_{2ij}^{\text{(}\text{k}\text{)}}$ and $\text{h}_{3ij}^{\text{(}\text{k}\text{)}}$ represent the lower, middle, and upper bounds, respectively. The fuzzy evaluations were transformed into crisp values using the CFCS (Converting Fuzzy data into Crisp Scores) method. The resulting crisp values were then aggregated across experts to construct the initial direct-relation matrix.

*Step 1: Construction of the Initial Direct-Relation Matrix*

The CFCS procedure was applied to convert triangular fuzzy numbers into crisp values through normalization, left–right score transformation, and defuzzification (Eqs. A1–A5).

(1) Normalization:

$\begin{matrix} \Delta_{\min}^{\max}=\max h_{3ij}^{\text{（}k\text{）}}-\min h_{1ij}^{\text{（}k\text{）}} \\ xh_{1ij}^{\text{（}k\text{）}}=(h_{1ij}^{\text{（}k\text{）}}-\min h_{1ij}^{\text{（}k\text{）}})/\Delta_{\min}^{\max} \\ xh_{2ij}^{\text{（}k\text{）}}=(h_{2ij}^{\text{（}k\text{）}}-\min h_{1ij}^{\text{（}k\text{）}})/\Delta_{\min}^{\max} \\ xh_{3ij}^{\text{（}k\text{）}}=(h_{3ij}^{\text{（}k\text{）}}-\min h_{1ij}^{\text{（}k\text{）}})/\Delta_{\min}^{\max} \end{matrix}$ (A1)

(2) Calculation of normalized left (***ls***) and right (***rs***) values:

$\begin{matrix} xls_{ij}^{\text{（}k\text{）}}=xh_{2ij}^{\text{（}k\text{）}}/(1+xh_{2ij}^{\text{（}k\text{）}}-xh_{1ij}^{\text{（}k\text{）}}) \\ xrs_{ij}^{\text{（}k\text{）}}=xh_{3ij}^{\text{（}k\text{）}}/(1+xh_{3ij}^{\text{（}k\text{）}}-xh_{2ij}^{\text{（}k\text{）}}) \end{matrix}$ (A2)

(3) Calculate the overall normalized crisp value:

$x_{ij}^{\text{（}k\text{）}}=[xls_{ij}^{\text{（}k\text{）}}(1-xls_{ij}^{\text{（}k\text{）}})+xrs_{ij}^{\text{（}k\text{）}}xrs_{ij}^{\text{（}k\text{）}}]/[1-xls_{ij}^{\text{（}k\text{）}}+xrs_{ij}^{\text{（}k\text{）}}]$ (A3)

(4) Calculation of crisp values for each expert questionnaire:

$\mu_{ij}^{\text{（}k\text{）}}=\min x_{1ij}^{\text{（}k\text{）}}+x_{ij}^{\text{（}k\text{）}}\times\Delta_{\min}^{\max}$ (A4)

(5) Integration of crisp values from all expert questionnaires:

$a_{ij}=\frac{1}{k}(\mu_{ij}^{1}+\mu_{ij}^{2}+\cdots+\mu_{ij}^{k})$ **.** (A5)

*Step* 2: *Construction of the Total Influence Matrix*

The normalized direct-relation matrix was denoted as $X$. The total influence matrix $T$ was derived using the DEMATEL infinite series formulation:

$X\text{=}\kappa\times A , \kappa=\max\left[\max_{i} \sum_{j=1}^{n} |a_{ij}| ,\max_{j} \sum_{i=1}^{n} |a_{ij}|\right]$ (A6)

$T\text{=}\left[t_{ij}\right]_{n\times n}=X+X^{2}+X^{3}+\cdots=\sum_{k=1}^{\infty} X^{k}=X(I-X)^{-1},$ (A7)

where $I$is the identity matrix of$n\times n$, and$X^{\infty}=\left[0\right]_{n\times n}$.

*Step 3: Construction of the Influence Network Relation Map (INRM)*

The prominence and relation values of each factor were obtained based on row and column sums of $T$:

$K=\left[k_{j}\right]_{n\times1}=\left[\sum_{i=1}^{n} t_{ij}\right]_{1\times n}^{T} , R=\left[r_{i}\right]_{n\times1}=\left[\sum_{j=1}^{n} t_{ij}\right]_{n\times1}.$ (A8)
