# Supplementary material for: Research on the emotional experience of online tourists in Zhangjiajie based on text mining and LDA-FDEMATEL
Source: PLoS One. 2026 Oct 1;21(10):e0355471. doi: 10.1371/journal.pone.0355471 (PMC13630242; doi:10.1371/journal.pone.0355471)
Supplement: Supplementary file 2 [file pone.0355471.s002.docx]

**Appendix B. Modified DANP Procedure**

*Step 1: Dimension-Level Weighting*

The initial influence matrix $A^{D}$at the dimension level was normalized as:

$X^{D}\text{=}\frac{A^{D}}{\kappa^{D}} , \kappa^{D}=\max\left[\max_{i} \sum_{j=1}^{n} |a_{ij}^{D}| ,\max_{j} \sum_{i=1}^{n} |a_{ij}^{D}|\right].$ (B1)

The total influence matrix of dimensions was computed as:

$T^{D}\text{=}\left[t_{_{ij}}^{D}\right]_{n\times n}=X^{D}+(X^{D})^{2}+(X^{D})^{3}+\cdots=X^{D}(I-X^{D})^{-1}.$ (B2)

Column normalization was then applied to derive the dimension weight matrix:

 (B3)

 (B4)

$W_{_{D}}=\left[T_{_{D}}^{\text{*}}\right]^{T}=\left[\begin{matrix} t_{11}^{_{D}^{*}} & \cdots& t_{i1}^{_{D}^{*}} & \cdots& t_{m1}^{_{D}^{*}} \\ \vdots& & \vdots& & \vdots\\ t_{1j}^{D^{*}} & \cdots& t_{ij}^{_{D}^{*}} & \cdots& t_{mj}^{D^{*}} \\ \vdots& & \vdots& & \vdots\\ t_{1m}^{_{D}^{*}} & \cdots& t_{im}^{D^{*}} & \cdots& t_{mm}^{_{D}^{*}} \end{matrix}\right].$ (B5)

The weights were obtained through iterative multiplication until convergence:

$(W_{D})^{\infty}=\lim_{k\to\infty} \left(W_{D}\right)^{k},$ (B6)

which yields stable dimension-level weights.

*Step 2. Construction of the Unweighted Supermatrix*

Let the total influence matrix of criteria be $T_{C}$, partitioned into $m\times m$blocks.

 (B7)

where $T_{pq}$reflects the influence of sub-criteria in dimension *p* on those in dimension *q*.

Each block was column-normalized as:

 (B8)

 (B9)

 (B10)

The normalized blocks form the unweighted supermatrix:

$W_{c}^{un}=(T_{c}^{\text{*}})^{\text{T}}=\left[\begin{matrix} T_{11}^{\text{*}} & \vdots& T_{i1}^{\text{*}} & \vdots& T_{m1}^{\text{*}} \\ \cdots& \vdots& \cdots& \vdots& \cdots\\ T_{1j}^{\text{*}} & \vdots& T_{ij}^{\text{*}} & \vdots& T_{mj}^{\text{*}} \\ \cdots& \vdots& \cdots& \vdots& \cdots\\ T_{1m}^{\text{*}} & \vdots& T_{im}^{\text{*}} & \vdots& T_{mm}^{\text{*}} \end{matrix}\right]\text{=}\left[\begin{matrix} W_{11}^{un} & \cdots& W_{1j}^{un} & \cdots& W_{1n}^{un} \\ \vdots& \ddots& \vdots& & \vdots\\ W_{i1}^{un} & \cdots& W_{ij}^{un} & \cdots& W_{in}^{un} \\ \vdots& & \vdots& \ddots& \vdots\\ W_{n1}^{un} & \cdots& M_{nj}^{un} & \cdots& W_{nn}^{un} \end{matrix}\right]$ (B11)

*Step 3. Construction of the Weighted Supermatrix*

The weighted supermatrix was obtained by integrating dimension-level weights:

$W_{c}=W_{D}\cdot W_{c}^{un}=\left[\begin{matrix} t_{11}^{_{dim}^{*}}\times T_{11}^{\text{*}} & \vdots& t_{i1}^{_{dim}^{*}}\times T_{i1}^{\text{*}} & \vdots& t_{m1}^{_{dim}^{*}}\times T_{m1}^{\text{*}} \\ \cdots& \vdots& \cdots& \vdots& \cdots\\ t_{1j}^{_{dim}^{*}}\times T_{1j}^{\text{*}} & \vdots& t_{ij}^{_{dim}^{*}}\times T_{ij}^{\text{*}} & \vdots& t_{mj}^{_{dim}^{*}}\times T_{mj}^{\text{*}} \\ \cdots& \vdots& \cdots& \vdots& \cdots\\ t_{1m}^{_{dim}^{*}}\times T_{1m}^{\text{*}} & \vdots& t_{im}^{_{dim}^{*}}\times T_{im}^{\text{*}} & \vdots& t_{mm}^{_{dim}^{*}}\times T_{mm}^{\text{*}} \end{matrix}\right].$ (B12)

This ensures that criterion weights reflect the relative importance of their parent dimensions.

*Step 4: Derivation of the Limiting Supermatrix*

The limiting supermatrix was obtained by raising the weighted supermatrix to sufficiently large powers until convergence:

$(W_{c})^{\infty}=\lim_{k\to\infty} \left(W_{c}\right)^{k}.$ (B13)

The resulting stable matrix provides the final global weights of emotional factors, capturing both direct and indirect influences as well as hierarchical dependencies.
