# Supplementary material for: Research on the emotional experience of online tourists in Zhangjiajie based on text mining and LDA-FDEMATEL
Source: PLoS One. 2026 Oct 1;21(10):e0355471. doi: 10.1371/journal.pone.0355471 (PMC13630242; doi:10.1371/journal.pone.0355471)
Supplement: Supplementary file 3 [file pone.0355471.s003.docx]

**Human Participants Research Checklist**

***Complete the following if your study involved human participants or human participants’ data. These questions should be addressed for prospective and retrospective studies.***

1. Did you obtain ethics approval for this study?
   - If yes, please upload (file type “Other”) the original approval document you received from your ethics committee. If the original document is in another language, please also provide an English translation.

___ Uploaded	_V _ N/A

- If you did not obtain ethical approval, please explain why this was not required below.

*Formal ethics approval was not obtained because, under the institutional requirements applicable at the time of data collection, this non-interventional and minimal-risk tourism management study did not require formal ethics committee review. The text-mining component used only publicly visible user-generated textual content collected from Ctrip, Tongcheng, and Dianping in compliance with the platforms’ terms of service. The study involved no direct interaction or intervention with the authors of the online reviews. During data preprocessing, all potential personal identifiers, including usernames, user IDs, profile information, hyperlinks, and other metadata, were removed, and no personally identifiable information was retained in the final dataset. The expert component involved only non-sensitive professional judgments collected through correspondence questionnaires. All experts participated voluntarily, and only those willing to participate completed and returned the questionnaire. No personally identifiable or sensitive information was collected from the experts, and their responses were anonymized, aggregated at the group level, and used exclusively for academic research.*

2. If you prospectively recruited human participants for the study – for example, you conducted a clinical trial, distributed questionnaires, or obtained tissues, data or samples for the purposes of this study, please report in the Methods:
   i. the day, month and year of the **start and end** of the recruitment period for this study.
   ii. whether participants provided informed consent, and if so, what type was obtained (for instance, written or verbal, and if verbal, how it was documented and witnessed). If your study included minors, state whether you obtained consent from parents or guardians. If the ethics committee waived the need for consent, please include this information.

_V Completed	___ N/A

*Seventeen adult experts were invited to participate through correspondence questionnaires between 1 November 2024 and 15 December 2024. Participation was entirely voluntary. The invited experts were informed of the study purpose, the intended academic use of their responses, and the confidentiality arrangements. Only experts who were willing to participate completed and returned the questionnaire; therefore, consent to participate was indicated by voluntary completion and return of the questionnaire. No minors participated in the study. As reported in Section 4.1 of the manuscript, no personally identifiable or sensitive information was collected, and all expert responses were anonymized and aggregated at the group level.*

3. If you are reporting a retrospective study of medical records or archived samples, please report in the Methods section:
i. the day, month and year when the data were accessed for research purposes
ii. whether authors had access to information that could identify individual participants during or after data collection

___ Completed	V	N/A

*N/A. This study did not involve a retrospective analysis of medical records, archived human samples, or clinical data.*
